# Longitudinal study of Middle East Respiratory Syndrome coronavirus infection in dromedary camel herds in Saudi Arabia, 2014–2015

**DOI:** 10.1038/emi.2017.44

**Published:** 2017-06-21

**Authors:** Maged Gomaa Hemida, Abdulmohsen Alnaeem, Daniel KW Chu, Ranawaka APM Perera, Samuel MS Chan, Faisal Almathen, Emily Yau, Brian CY Ng, Richard J Webby, Leo LM Poon, Malik Peiris

**Affiliations:** 1 Department of Microbiology and Parasitology, College of Veterinary Medicine, King Faisal University, Alhufuf, Al-Ahsa 31982, Saudi Arabia; 2 Department of Virology, Faculty of Veterinary Medicine, Kafrelsheikh University, Kafrelsheikh 33516, Egypt; 3 Department of Clinical Studies, College of Veterinary Medicine, King Faisal University, Alhufuf, Al-Ahsa 31982, Saudi Arabia; 4 School of Public Health, The University of Hong Kong, Hong Kong, China; 5 Department of Public Health and Animal Welfare, College of Veterinary Medicine, King Faisal University, Alhufuf, Al-Ahsa 31982, Saudi Arabia; 6 Division of Virology, Department of Infectious Diseases, St Jude Children’s Research Hospital, Memphis, TN 38105, USA

**Keywords:** camel, cohort, coronavirus, dromedary, immunity, MERS coronavirus, reinfection

## Abstract

Two herds of dromedary camels were longitudinally sampled with nasal and rectal swabs and serum, between September 2014 and May 2015, and the samples were tested for Middle East Respiratory Syndrome (MERS) coronavirus RNA and antibodies. Evidence of MERS-CoV infection was confirmed in one herd on the basis of detection of virus RNA in nasal swabs from three camels and significant increases in the antibody titers from three others. The three viruses were genetically identical, thus indicating introduction of a single virus into this herd. There was evidence of reinfection of camels that were previously seropositive, thus suggesting that prior infection does not provide complete immunity from reinfection, a finding that is relevant to camel vaccination strategies as a means to prevent zoonotic transmission.

## INTRODUCTION

Middle East Respiratory Syndrome coronavirus (MERS-CoV) was initially identified in Saudi Arabia in 2012.^1^ As of 5 December 2016, there were over 1800 laboratory-confirmed cases.^2^ Camels are known to be the natural host for MERS-CoV and the source of zoonotic infection.^3, 4, 5^ Zoonotic transmission events may be mild and unrecognized but may lead to transmission between humans, thus leading to MERS outbreaks in health care facilities.^6, 7^ The SARS epidemic of 2003 was heralded by repeated small zoonotic outbreaks in 2002 that were self-limited until a strain of SARS CoV that was well adapted to humans emerged and led to a global epidemic that affected approximately 8000 patients in 25 countries across five continents.^8^ Given this demonstration of the capacity for novel coronaviruses to emerge from animals to cause major outbreaks in humans, the threat from MERS-CoV remains a cause for global health concern.

Vaccination of dromedary camels has been proposed as a means to reduce the threat of zoonotic MERS.^9^ It is therefore important to establish the epidemiology of MERS-CoV transmission within camels, and especially whether prior infection protects against subsequent reinfection. We therefore carried out a longitudinal study of two camel herds in the Kingdom of Saudi Arabia to elucidate MERS-CoV infection and transmission.

## MATERIALS AND METHODS

### Sample collection

Nasal and rectal swabs and serum samples were collected from dromedary camels in two herds in the Eastern and Central regions of Saudi Arabia between September 2014 and May 2015. The same animals were resampled whenever possible. The ages of the animals were assessed on the basis of farm records and, when records were not available, by examination of dentation. Swab samples were collected in viral transport medium and stored at −80 °C.

#### Herd 1

This group was a closed camel herd of ~80 animals in the Eastern Province. The camels were housed in one compound and provided with feed in barns. There was no contact with nomadic camel herds. Occasionally, animals purchased from outside (for example, camel markets) may be introduced into the herd. A previous study of this camel herd has been published.^10^

#### Herd 2

This group was a camel herd of ~100 dromedaries in the Central Province. The animals were held in one barn and were separated into several subgroups (males, pregnant and lactating animals). The different animal groups were separated within the same compound by only a fence. Although this herd was largely closed, animals purchased from local markets are occasionally introduced to the herd. Nomadic camel herds of Bedouins graze in the surrounding area, especially in the fall and winter. Other animals, such as sheep and goats, are also sometimes present in the same area. Stray dogs, foxes, rodents and birds such as doves and crows are also frequently seen in proximity to this herd.

### RT-PCR and serology testing

The total nucleic acid extracted from the swabs was tested for MERS-CoV RNA by using reverse transcription PCR (RT-PCR). An RT-PCR assay targeting the region upstream of the envelope protein gene (upE) was used for screening, and the confirmation of any screen-positive samples was done using RT-PCR that targeted open reading frame 1a (ORF-1a).^4, 11^ A 7675 nucleotide (nt) region of the genome from the spike gene to the N gene (22 140–29 814 nt in the reference MERS-EMC strain sequence) was RT-PCR amplified as overlapping PCR amplicons and sequenced by Sanger sequencing from any RT-PCR-positive samples.

Evidence of other coronaviruses was sought by testing the swab samples using a pan-coronavirus-nested PCR that was targeted at the conserved RNA-dependent RNA polymerase (RdRp) gene of coronaviruses that we have previously developed and reported.^4, 12^

MERS-CoV antibody was tested using a validated MERS-CoV spike pseudoparticle neutralization test (ppNT) as previously described.^13^

## RESULTS

In herd 1, 29 camels, including 2 calves, were sampled; 8 were sampled in October 2014, 9 in November 2014, 15 in January 2015 and 13 in February 2015. Animals had both swabs and serum collected, with the exception of one animal in November, one in January and one in February. The MERS-CoV RNA was not detected in any of the camels sampled from herd 1 (Table 1). All the camels that were sampled had MERS-CoV antibody, thus indicating past infection.

In herd 2, 70 dromedaries were sampled, including 16 calves ⩽2 years of age, 5 animals 3–5 years of age, 35 animals ⩾6 years of age and 14 animals of undetermined age. Ten camels were sampled in September 2014, 43 in October 2014, 28 in November 2014, 45 in January 2015, 24 in February/March 2015 and 52 in May 2015. The number of swabs and sera collected at each sampling occasion is detailed in Table 2. The results are summarized in Table 3. Both nasal and rectal swabs were collected, except where indicated in Table 2.

MERS-RNA was detected in nasal swabs from three dromedaries in November 2014 (Tables 2 and 3), thus suggesting that this herd was infected with MERS-CoV. The corresponding rectal swabs were RT-PCR negative. The viral load in these three nasal swab specimens was 2.2 × 10^3^ copies per mL (specimen F2-3); 0.7 × 10^3^ copies per mL (specimen F2-33) and 1.4 × 10^3^ copies per mL (specimen F2-51) (Table 4). These three animals were seropositive in the sampling carried out in November and did not demonstrate fourfold increases in their antibody titers after infection (Tables 2 and 4). None of the rectal swabs were positive for MERS-CoV. MERS-CoV antibody titers in animals with sequential serum samples showed three other animals with significant (fourfold or greater) increases in antibody titers, thus suggesting MERS-CoV infection in three additional animals in the periods September–October 2014 (animal F2-26), November 2014–January 2015 (animal F2-34) and March–May 2015 (animal F2-5) (Tables 2 and 4). These results suggested that virus transmission occurred during the September–October 2014 period and continued through to March–May 2015.

**Figure 1:**
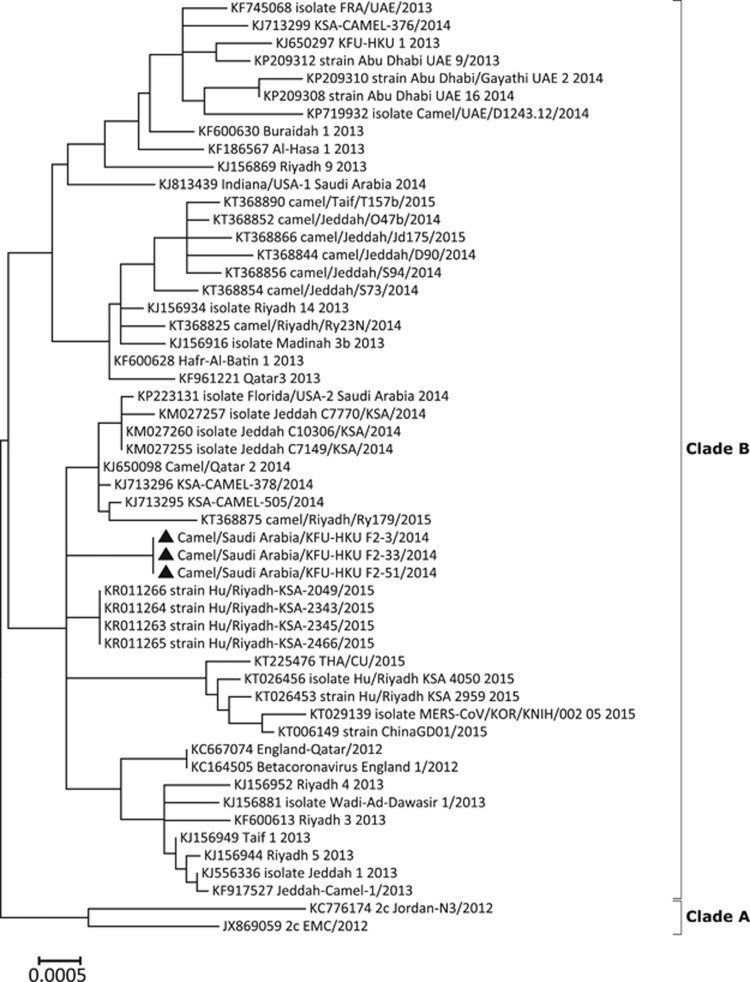
Phylogenetic analysis of MERS-CoV found in three RT-PCR-positive camels in herd 2 in November 2014 using MEGA6. Gene sequences of 7675 nucleotides from the MERS-CoV genome region spanning from the spike gene to the N gene (corresponding to nucleotide positions 22 140–29 814 of MERS-CoV/EMC/2012) were aligned using MUSCLE. The evolutionary history of the phylogenetic tree was inferred using the maximum likelihood method based on the Tamura-Nei model. Sequences from the current study are indicated by solid triangle.

A 7675 nt region of the genome from the spike gene to the N gene was sequenced from the three positive samples (GenBank accession numbers KY706245–KY706247). These three sets of sequences were found to be identical to each other, thus suggesting that a single virus had been introduced into this herd. The sequences were closely related but not genetically identical to the MERS-CoV strain Riyadh/Riyadh179/2015 (GenBank accession no.: KT368875), which has been previously reported in dromedaries in Saudi Arabia (Figure 1). In addition, a 1164 nt region of the N gene (28 650–29 814 nt of MERS-CoV genome) was sequenced from these three viruses and were found to be identical to each other. Phylogeny also confirmed that these viruses were identical and closely related to Riyadh/Riyadh179/2015 (data not shown).

Pan-coronavirus RT-PCR detecting most known (and likely unknown) coronaviruses was negative.

## DISCUSSION

Our longitudinal study on two dromedary herds demonstrated high levels of seropositivity in both herds, a result in agreement with findings from many other studies reporting high seroprevalence in adult animals in the Arabian Peninsula and Africa.^3, 4, 13, 14, 15, 16, 17^ One of these herds, namely, herd 2, had evidence of an active MERS-CoV infection. The RT-PCR detection of MERS-CoV confirmed the infection of three camels in November 2014, and viral molecular epidemiology confirmed that these three infections most probably arose from introduction of one virus into the herd. The three animals that were RT-PCR positive for MERS-CoV in November 2014 were seropositive in the previous serum sampling in October but were reinfected in spite of prior moderate (titer 1:320) or high (1:40 960) antibody titers. Because these animals were 2 years of age, the serum antibody that was detected prior to infection would have resulted from infection after birth rather than being antibody passively acquired from the dam. Given the camels’ age, such natural infections had occurred within the past 2 years.

A previous study of another infected camel herd has shown infection of both adults and calves.^10^ Because adult animals (which are very likely to be seropositive) and a 2-week-old calf (which was very likely to have maternal antibodies) were found to be infected, it was presumed that neither prior natural infection nor passive antibodies would produce sterilizing immunity.^10^ However, there was no conclusive evidence of antibody detected prior to infection. The lack of correlation between the viral RNA loads and the levels of neutralizing antibody in camels slaughtered in an abattoir in Qatar has also been taken to indicate that prior antibodies may not provide sterilizing immunity.^17^ However, again, conclusive evidence of the presence of antibodies before infection was not available. In contrast, a study of infection within camel herds whose dam and calf pairs were studied, has reported that infection occurred in the calves but not in the seropositive dams, thus suggesting that prior immunity in older animals may prevent reinfection.^16^ In another study in which 11 calves were prospectively followed up during the first year of life, five calves were found to be infected within the first month of life even though they had evidence of passively acquired maternal antibodies, thus suggesting that the maternal antibodies do not provide complete protection from reinfection.^18^ Evidence of reinfection of adult seropositive camels was also reported in a longitudinal study of camels in Egypt.^19^

The present study provides conclusive evidence that reinfection of previously seropositive camels can occur. This observation has important implications for the feasibility of using vaccination of camels as a means to control MERS-CoV transmission within camel herds with the aim of reducing zoonotic transmission. Reinfection in previously seropositive animals may occur because MERS-CoV infection in camels is a mucosal infection and the serum antibody might not be an accurate predictor of the effective mucosal antiviral immunities that can provide sterilizing immunity. None of the studies to date, including our own, have tested for evidence of mucosal IgA immunity in the oral or nasopharyngeal cavity, and this deficiency remains a crucial gap in the understanding of protection from reinfection.

The lack of protection against natural reinfection in the field thus raises questions about the potential duration of protection conferred by MERS-CoV infection or vaccines. Previous experimental studies of camels vaccinated with a vaccinia-vectored MERS-CoV vaccine that were challenged 3 weeks later with a dose of 10^7^ TCID_50_ MERS-CoV inoculated intra-nasally showed a decrease, but not a complete elimination of detectable virus RNA or infectious virus.^9^ However, this test was an experimental challenge with what may be a non-physiological dose of virus and after a very short post-vaccination interval. These conditions may not reflect the situation in the field. Experimentally infected alpacas have been found to be protected against MERS-CoV reinfection; again, this study involved a challenge that occurred at a short interval after the previous infection.^20^

In contrast to data from the experimental challenge of vaccinating animals, our studies have merit in that the infecting dose leading to infection was physiologically relevant to the field conditions and that the challenge probably occurred many months or years after the initial infection. The viral load in nasal swabs in the three RT-PCR-positive specimens was low and ranged from 0.7 × 10^3^ to 2.2 × 10^3^ copies per mL. In contrast, the viral loads in camels in our previously reported study of transmission within a camel herd ranged from 3.3 × 10^3^ to 1.78 × 10^8^ copies per mL. It is not clear whether the low viral load observed in the reinfected animals in herd 2 would be sufficient to result in onward transmission to other animals.

In addition to the three RT-PCR-positive camels, the serology data confirmed evidence of infection in three other camels from as early as September–October 2014 and confirmed that the infection was active until the November 2014–January 2015 period. Whether these infections were also a result of the same virus introduction cannot be confirmed, but this possibility is likely. If this were the case, a single introduction of MERS-CoV would probably have led to virus circulation in herd 2 from September 2014 until at least November 2014, a period of 2–3 months. In a previous study of herd 1, we have demonstrated virus circulation for at least 1 month, November–December 2013.^10^ Data from this study showed that camels in herd 2 that were RT-PCR positive did not demonstrate a fourfold increase in antibody titer, although two camels showed a non-significant twofold increase in titers. Thus, we cannot exclude infections in other sampled animals whose antibody titers were not observed to rise by fourfold, the usual criterion for a significant antibody response. Because of these observations, and because all animals in herd 2 were not sampled, our detection of six infected camels is likely to be an underestimate of the extent of transmission within this herd.

Age data were available for 56 of the sampled camels in herd 2. Among those animals with a known age, 5 (83.3%) of the 6 camels with evidence of MERS-CoV infection, either by RT-PCR or serological tests, were aged 2 years or younger, whereas only 11 (22.8%) of the 50 MERS-CoV-negative animals were of similar age (Fisher’s test *P*=0.0056). This result suggested that younger camels are more likely to acquire infection within an infected herd, as has been previously reported.^16^

A limitation of the present study was that the viral load data were available at only one point in time during the time course of infection, because the interval between specimens was ⩾1 month. Furthermore, specimens were not collected every month from all the animals that were followed up. Information on mucosal antibody titers would have been very useful, but it is known that antibodies that are functional (for example, neutralizing) against viruses are very difficult to accurately assay because of the presence of many non-antibody-mediated mechanisms for virus neutralization in saliva.

Another limitation in our study was that our neutralization tests were done with MERS from the EMC strain rather than a contemporary MERS-CoV strain. However, we and others have shown that genetically diverse MERS-CoV are antigenically homogenous in cross-neutralization tests carried out by conventional microneutralization or pseudotype neutralization tests.^10, 21^ Thus, virus antigenic variation was unlikely to confound our serological tests or lead to antigenic escape from prior antibody immunity.

In conclusion, we demonstrate the reinfection of camels that were previously seropositive with very high serum antibody titers. It may be possible that such reinfections are associated with lower viral loads and may be less transmissible to other animals, but this hypothesis remains to be confirmed. Well-designed field studies of vaccinated animals followed up over a period of longer than 1 year would be required to assess the effects of vaccination on decreasing virus transmission between camels. Longitudinal studies of dromedary herds such as those reported here provides useful data for the design of such systematic studies.

## Figures and Tables

**Table 1:** Dromedary herd 1: specimens collected and MERS-CoV RT-PCR and screening antibody test results

	**October 2014**		**November 2014**		**January 2015**		**February 2015**	
	**RT-PCR positive**	**Antibody positive**^a^	**RT-PCR positive**	**Antibody positive**	**RT-PCR positive**	**Antibody positive**	**RT-PCR positive**	**Antibody positive**
No. of animals tested	8	8	9	8	15	15	13	12
Sex	F=7; M=1		F=7; M=2		F=11; M=4		F=10; M=3	
Age (years)	2, 5, 9, 9, 10, 11, 13, 14		0.7,0.8, 0.8, 3, 4, 4, 4, 8, 8		0.3, 0.5, 1.5, 3, 3, 4, 4, 4, 5, 6, 8, 10, 12, 13, 14		1, 3, 3, 4, 4, 4, 4, 5, 6, 10, 12, 13, 13	
No. of positive	0	8	0	8	0	14	0	12
% Positive	0	100	0	100	0	100	0	100

a MERS-CoV antibody positive at a screening dilution of 1:20.

**Table 2:** Samples of camels collected and MERS-CoV RT-PCR and serological results

**Animal**			**September 2014**		**October 2014**		**November 2014**		**January 2015**		**February/March 2015**		**May 2015**	
**ID no.**	**Sex**	**Age (years)**	**RNA**	**Titer**	**RNA**	**Titer**	**RNA**	**Titer**	**RNA**	**Titer**	**RNA**	**Titer**	**RNA**	**Titer**
F2-1	F	23			NA	2560	NEG	2560	NEG	2560	NEG	2560	NEG	2560
F2-2	F	2			NEG	640	NEG	320	NEG	320			NEG	320
F2-3	F	2			NA	2560	POS (N)	5120						
F2-4	F	20			NA	1280	NEG	2560	NEG	1280	NEG	1280	NEG	640
F2-5	F	1									NEG	160	NEG	640
F2-6	F	6							NEG	1280	NEG	1280	NEG	2560
F2-7	M	1									NEG	1280	NA	320
F2-8	F	17			NA	20480	NEG	10240	NEG	20480	NEG	10240	NEG	10240
F2-9	F	5											NEG	NA
F2-10	F	13			NEG	2560	NEG	2560	NEG	1280			NEG	1280
F2-11	F	17			NA	640	NEG	640	NEG	640	NEG	640	NEG	640
F2-12	F	12			NEG	1280	NEG	1280	NEG	1280			NEG	2560
F2-13	F	20			NA	320	NEG	640	NEG	320	NEG	320	NEG	320
F2-14	F	7			NA	10240	NEG	5120	NEG	2560	NEG	5120	NEG	5120
F2-15	F	6											NEG	640
F2-16	F	2			NA	1280	NEG	1280	NEG	2560				
F2-17	F	12.5			NA	640	NEG	640	NEG	640	NEG	320	NEG	320
F2-18	F	8			NA	640	NEG	320	NEG	640	NEG	640	NEG	320
F2-19	F	13	NEG	640	NA	1280			NEG	1280	NEG	1280	NEG	1280
F2-20	F	9	NEG	NA	NA	1280	NA	2560	NEG	2560	NEG	1280	NEG	1280
F2-21	F	7			NEG	NA			NEG	10240			NEG	5120
F2-22	F	1.5			NEG	NA							NEG	NA
F2-23	F	14	NEG	20480	NEG	10240			NEG	10240				
F2-24	F	1							NEG	320				
F2-25	F	20									NA	2560		
F2-26	F	8	NEG	320	NEG	1280			NEG	2560			NEG	1280
F2-27	F	16			NA	1280	NEG	640	NEG	640	NEG	1280	NEG (NO)	640
F2-28	M	10											NEG (RO)	NA
F2-29	F	8			NEG	2560	NEG	5120	NEG	2560			NEG	1280
F2-30	F	1.1							NEG	1280				
F2-31	F	6			NEG	NA			NEG	2560				
F2-32	F	2			NEG	10240	NEG	10240	NEG	5120			NEG	10240
F2-33	M	2			NEG	40960	POS (N)	40960	NEG	40960			NEG	20480
F2-34	F	2			NEG	320	NEG	320	NEG	1280			NEG	1280
F2-35	NA	NA									NA	640		
F2-36	F	3			NEG	NA			NEG	640			NEG	320
F2-37	M	NA			NEG	NA							NEG	320
F2-38	NA	NA			NEG	NA							NEG	NA
F2-39	F	17	NEG	2560	NA	1280								
F2-40	F	15							NEG	320	NEG	640	NEG	320
F2-41	F	15	NEG	1280	NA	640			NEG	640	NEG	640	NEG	640
F2-42	NA	NA											NEG (RO)	10240
F2-43	F	3			NEG	NA			NEG	640			NEG	320
F2-44	F	14	NEG	640	NEG	640			NEG	640			NEG	320
F2-45	M	0.75							NEG	80				
F2-46	F	11			NEG	2560	NEG	2560	NEG	NA	NEG	2560	NEG	2560
F2-47	NA	NA											NEG	NA
F2-48	F	8			NEG	NA			NEG	640			NEG	320
F2-49	F	17							NEG	10240	NEG	2560	NEG	2560
F2-50	F	19							NEG	NA				
F2-51	F	2			NEG	320	POS (N)	320	NEG	640	NA	320	NEG	NA
F2-52	F	3			NEG	2560	NEG	NA	NEG	1280			NEG	2560
F2-53	F	5					NEG	NA	NEG	2560	NEG (RO)	2560	NEG	2560
F2-54	NA	NA											NEG	NA
F2-55	F	11	NEG	NA			NA	1280	NEG	640	NEG	640		
F2-56	F	6					NEG	1280						
F2-57	F	2			NEG	NA							NEG	320
F2-58	F	16	NEG	640	NEG	640			NEG	640			NEG	320
F2-59	M	0.7							NEG	320				
F2-60	F	10	NEG	5120	NA	2560			NEG	2560	NEG	5120	NEG	5120
F2-61	F	8			NEG	10240	NEG	10240	NEG	10240			NEG	5120
F2-62	M	0.5							NEG	5120				
F2-63	NA	NA			NEG (NO)	2560								
F2-64	NA	NA			NA	2560	NEG	1280						
F2-65	NA	NA			NA	1280	NEG	1280						
F2-66	NA	NA											NEG	2560
F2-67	NA	NA											NEG	320
F2-68	NA	NA											NEG	5120
F2-69	NA	NA											NEG	320
F2-70	NA	NA											NEG	NA

Abbreviations: nasal swab, N; specimen not collected, NA; only nasal swab tested, NO; only rectal swab tested, RO.

**Table 3:** Dromedary herd 2: specimens collected and summary results of MERS-CoV RT-PCR, antibody screening and rising antibody titers

	**September 2014**		**October 2014**		**November 2015**		**January 2015**		**February–March 2015**		**May 2015**	
	**RT-PCR**	**Antibody positive or rising antibody titers**^a^	**RT-PCR**	**Antibody positive or rising antibody titers**^a^	**RT-PCR**	**Antibody positive or rising antibody titers**^a^	**RT-PCR**	**Antibody positive or rising antibody titers**^a^	**RT-PCR**	**Antibody positive or rising antibody titers**^a^	**RT-PCR**	**Antibody positive or rising antibody titers**^a^
No. of animals tested	10	8	25	34	26	26	45	43	21	24	51	44
No. of RNA positive	0	NR	0	NR	3	NR	0	NR	0	NR	0	NR
No. of sero positive	NR	8	NR	34	NR	26	NR	43	NR	24	NR	44
No. of rise in antibody titers	NR	NR	NR	1	NR	0	NR	1	NR	0	NR	1

Abbreviation: not relevant, NR.

a MERS-CoV antibody positive at a screening dilution of 1:20; fourfold rise in antibody titer.

**Table 4:** Dromedary herd 2: Viral load and reciprocal antibody titers of the six animals with evidence of MERS-CoV infection

**Animal ID number**	**Age (years) and sex**	**September 2014**		**October 2014**		**November 2015**		**January 2015**		**February-March 2015**		**May 2015**	
		**RT-PCR**	**Antibody titers**	**RT-PCR**	**Antibody titers**	**RT-PCR positive (viral load genome copies per mL)**	**Antibody titers**	**RT-PCR**	**Antibody titers**	**RT-PCR**	**Antibody titers**	**RT-PCR**	**Antibody titers**
F2-3	2, F	NA	NA	NA	2560	Pos (2.2 × 10^3^)	5120	NA	NA	NA	NA	NA	NA
F2-33	2, M	NA	NA	Neg	40960	Pos (0.7 × 10^3^)	40960	Neg	40960	NA	NA	Neg	20480
F2-51	2, F	NA	NA	Neg	320	Pos (1.4 × 10^3^)	320	Neg	640	NA	320	Neg	NA
F2-26	8, F	Neg	320	Neg	1280			Neg	2560	NA	ND	Neg	1280
F2-34	2, F	NA	NA	Neg	320	Neg	320	Neg	1280	NA	NA	Neg	1280
F2-5	1, F	NA	NA	NA	NA	NA	NA	NA	NA	Neg	160	Neg	640

Abbreviation: specimen not available, NA.
